# Hepatobiliary-pancreatic surgery for patients with a prepancreatic postduodenal portal vein: a case report and literature review

**DOI:** 10.1186/s12893-022-01508-z

**Published:** 2022-02-13

**Authors:** Taku Higashihara, Yasuhiro Morita, Tatsuya Hayashi, Makoto Takahashi, Norikazu Yogi, Shu Sasaki, Daren Zhou

**Affiliations:** Department of Surgery, Tokyo Metropolitan Tama Medical Center, 2-8-29, Musashidai, Fuchu, Tokyo, Japan

**Keywords:** Pancreaticoduodenectomy, Anomalous portal vein, Prepancreatic postduodenal portal vein, Case report, Hepatobiliary-pancreatic Surgery

## Abstract

**Background:**

Prepancreatic portal vein (PPV) is a congenital anatomical variant of the portal vein (PV). PPVs are extremely rare and generally classified into two categories, prepancreatic preduodenal portal vein and prepancreatic postduodenal portal vein (PPPV). Prepancreatic preduodenal portal veins are rare, with approximately 100 reported cases globally; PPPVs are even more atypical, with less than 20 documented cases globally. Despite the extremely low occurrence, PPPV knowledge and recognition are important, especially for hepatobiliary-pancreatic (HBP) surgeries, such as pancreaticoduodenectomy (PD) for patients of a PPPV. Here, we report a case of PPPV and a literature review.

**Case presentation:**

A 73-year-old-male with ampullary carcinoma underwent PD at our hospital. Preoperative enhanced CT revealed an abnormal L-shaped PV, identified as a PPPV. Both the PPPV and the postpancreatic “normal” superior mesenteric vein (SMV) divaricated from the SMV at the caudal side of the pancreas. A splenic vein and inferior mesenchymal vein flowed into the postpancreatic “normal” PV, which encircled the common bile duct and potentially flowed into the liver, forming a cavernous transformation at the hilar plate. During surgery, we attempted to isolate the PV from the pancreas and common bile duct. However, it was difficult to isolate from the pancreas. The PPPV was so fragile that bleeding from the PPPV became uncontrollable. To remove the tumor, we resected the PPPV and reconstructed a “normal” PV as an autogenous graft. To maintain intraoperative hepatic blood flow and avoid small bowel congestion, an antithrombogenic bypass catheter was placed between the SMV and umbilical vein during reconstruction. After surgery, several complications occurred, such as PV thrombosis and hyperammonemia. The patient was discharged on postoperative day 45.

**Conclusions:**

PPPV is a rare vascular variant but is easily diagnosed preoperatively due to its distinct shape on CT imaging. However, isolating the PPPV from the pancreas and bile duct is incredibly difficult and potentially associated with increased operative risks and postoperative complications. PV resection rather than isolation is a potential solution to reduce the risk of hemorrhage, even in the absence of invasion.

## Background

Awareness of vascular variants is essential to minimize postoperative risks associated with surgery, especially hepatobiliary-pancreatic (HBP) surgery. Anatomical variants of the branching pattern of the intrahepatic portal vein (PV) have been reported in approximately 20–35% of the population [1, 2]. However, congenital malformations, such as agenesis of the PV, preduodenal PV, and duplication of the PV, are less frequently reported [3]. Here, we have described a specific case of pancreaticoduodenectomy (PD) in a patient with an extremely rare congenital malformation a prepancreatic postduodenal portal vein (PPPV), in accordance with the CARE Guidelines [4]. Though unnecessary PV resection should not be considered because of the possibility of postoperative complications, it could bring benefits for the patients with PPPV who require HBP surgery.

## Case presentation

A 73-year-old Japanese male was transferred to our hospital with high levels of serum AST, ALT, and γ-GTP. He did not complain of any symptoms. His surgical history revealed that subtotal gastrectomy with Billroth II reconstruction was performed due to a gastric ulcer 33 years prior (the operation record was unavailable). His medical history also included multiple cerebral infarctions and a chronic subdural hematoma. There were no other significant findings in his family medical history, social history, or lifestyle history.

**Fig. 1:**
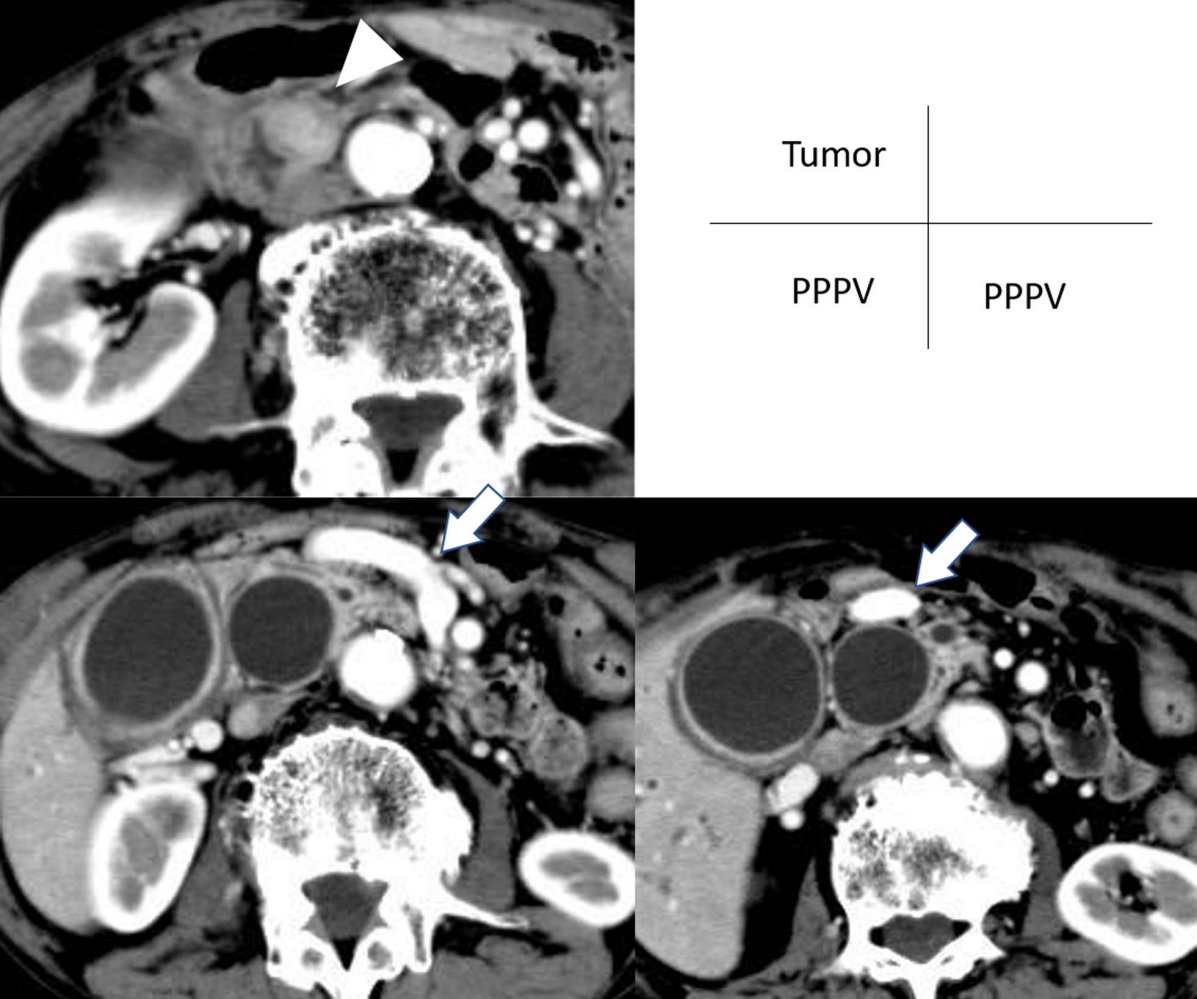
Contrast-enhanced CT image. **A** A tumor in the ampulla of Vater with low contrast was detected (arrowhead). **B** A PPPV (arrow) were divaricated from the SMV and crossed in front of the pancreas. **C** The PPPV crossed in front of the dilated common bile duct

Contrast-enhanced CT revealed a 16-mm tumor in the ampulla of Vater with low contrast (Fig. 1A). Despite no clinical signs of jaundice, both the main pancreatic duct and common bile duct were dilated. Due to obstruction of the bile duct and past Billroth II reconstruction, a double-balloon endoscopy-assisted endoscopic retrograde cholangiography was performed and revealed an exposed protruding tumor at the ampulla of Vater. Subsequent endoscopic biopsy revealed adenocarcinoma. Preoperative imaging showed no obvious evidence of vascular invasion or distant metastasis. Taken together, the ampullary carcinoma was diagnosed as stage IB, T2N0M0 (American Joint Committee on Cancer (AJCC), 8th edition [5].

**Fig. 2:**
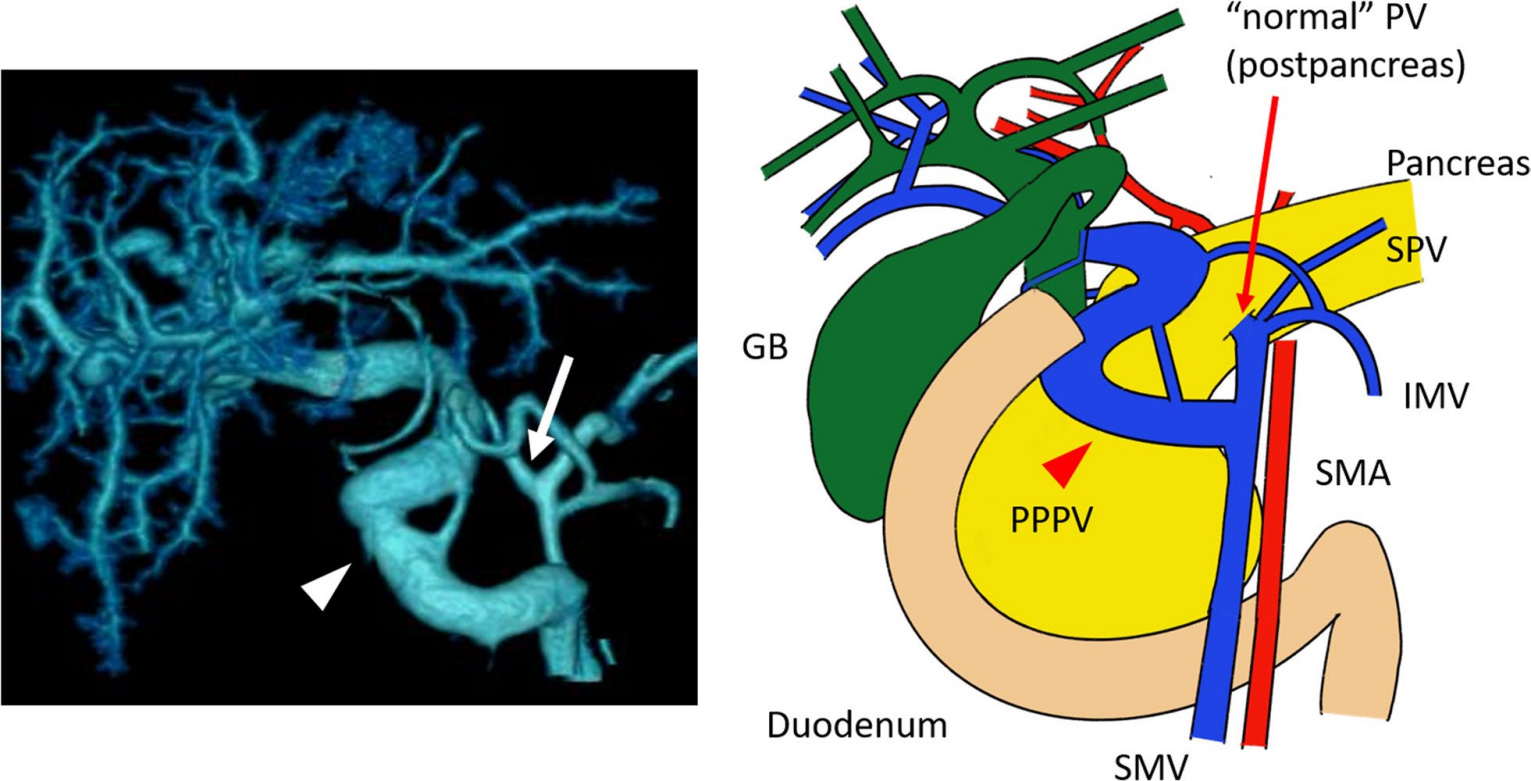
Three-dimensional angiography and its schema. The L-shaped thick vessel represents the PPPV (arrowhead). From the SMV, the thin “normal” PV was divaricated (arrow)

In addition, contrast-enhanced CT revealed abnormal branches of the PV (Fig. 1B, C). Both the “abnormally dilated PV” and the postpancreatic superior mesenteric vein (SMV) were divaricated from the SMV at the caudal side of the pancreas. A splenic vein (SPV) and inferior mesenteric vein flowed into the “normal” PV. The “normal” PV encircled the common bile duct and flowed into the S4 segment of the liver, forming a cavernous transformation at the hilar plate. The abnormal PV crossed in front of the pancreas and common bile duct and then entered the cavernous transformation behind the common hepatic duct. The bulbus was already resected when gastrectomy was performed; therefore, the relationship between the abnormal PV and duodenum was not obvious. Additionally, due to the cavernous transformation at the hilar plate, the branch to the right/left portal branches could not be detected. The dilated PV was diagnosed as a PPPV based on the characteristic L-shaped PV in three-dimensional angiography (Fig. 2).

**Fig. 3:**
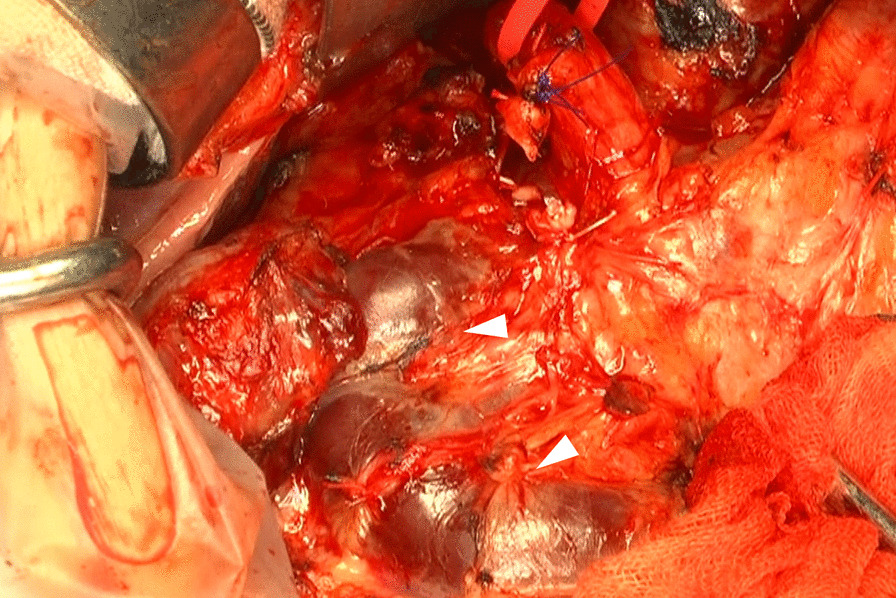
Intraoperative image (after gastric duodenal artery ligation). The boundary among the pancreas, duodenum, and PPPV (arrowhead) could not be clearly established

During the operation (Fig. 3), we attempted to isolate the PPPV from the bile duct and pancreas. However, the boundary between the PPPV and adjacent organs could not be clearly established. We tried to isolate the PPPV from the adjacent organs, but it was easily injured and bled if any load was applied because of its fragile wall. Even though we stopped the bleeding by suturing the hemorrhagic spot, each time isolation was attempted, rebleeding occurred. The attempt resulted in uncontrollable blood loss and necessitated PPPV resection to remove the ampulla of Vater and control the hemorrhage. An Anthron bypass catheter (Toray Medical Co., Ltd. Tokyo, Japan) was placed between the SMV and umbilical portion to avoid congestion of small intestine. Resection and reconstruction of PPPV was conducted using the postpancreatic “normal” PV as an autogenous graft. The PV clumping time was 116 min. After reconstruction, the intrahepatic PV flow was confirmed by ultrasound. The operating time was 16 h and 43 min, and operative blood loss was 13,994 ml.

Even though there is a lack of scientific evidence, unfractionated heparin (10,000 units/day) was administered on postoperative days 3–7 to prevent PV thrombosis. However, enhanced CT on postoperative day 8 showed thrombosis in the reconstructed PV. Fortunately, intrahepatic PV flow was favorable due to cavernous transformation. Then, edoxaban (30 mg/day) was administered starting on postoperative day 8. Several complications occurred but were treatable, such as hyperammonemia (treated by Lactulose and L-arginine/L-citrulline), pancreatic fistula (treated by abdominal drainage for 20 days) and delayed gastric emptying (treated by dietary manipulation and prokinetic medications such as metoclopromide and mosapride Citrate; 15 days after operation, his symptoms due to delayed gastric emptying were improved). The patient was discharged after 45 days of care.

**Fig. 4:**
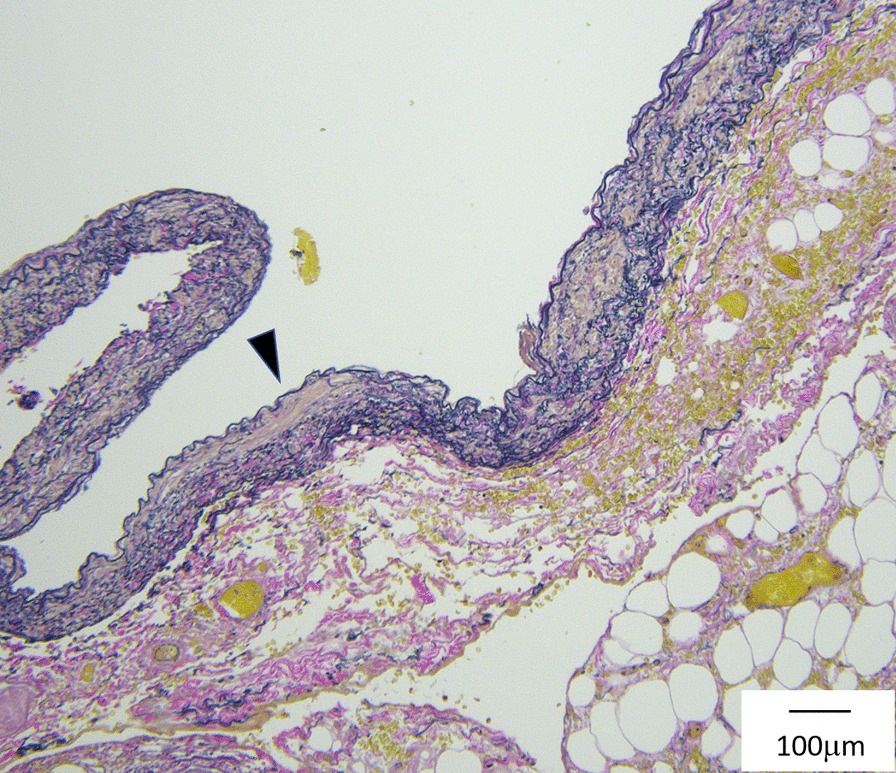
Pathological findings of the PPPV (EVG staining). The wall (arrowhead) was approximately 0.1 mm thick. The elastic fiber was partially sparse

In terms of pathological findings, the ampullary carcinoma was diagnosed as stage IIB (T2N1M0, AJCC, 8th edition [5]). The resected PPPV had a heterogeneous thickness and had much thinner walls (0.1–0.2 mm) than normal portal veins (1.6 ± 0.3 mm [6]) (Fig. 4). Additionally, the elastic fiber that formed the PV wall was partially sparse. There was no obvious finding for PV invasion. Twelve months after the operation, the patient is still alive without any recurrence.

## Discussion and conclusions

A wide range of congenital conditions may affect the portal venous system. A PPPV is an extremely rare congenital vascular variant and has a very complex embryological etiology. At the fourth week of embryonic life, three anastomoses develop around the duodenum and between the paired vitelline veins: cranial-ventral (within the liver), dorsal (posterior to the duodenum) and caudal-ventral (anterior to the pancreas). Under normal conditions, the ventral bud fuses side-by-side with the dorsal bud. The dorsal bud forms the upper head, body, and tail of the pancreas, whereas the ventral bud forms the inferior head and uncinated process [7, 8]. During normal embryogenesis, the dorsal pancreatic bud lies anterior to the left vitelline vein, resulting in a PV that is posterior to the pancreas and duodenum. However, in the case of a PPPV, the dorsal pancreatic bud is positioned posterior to the left vitelline vein moving the portal vein anterior to the pancreas and posterior to the duodenum [9]. This positioning might make it difficult to isolate PPPV from the pancreas.

A PPPV is often detected as an L-shaped or inverted L-shaped PV by contrast-enhanced CT [9, 10]. Additionally, PPPV cases often exhibit early PV branches or cavernous transformations around the portal hepatis or a junction between the SMV and SPV [11]. These features allow for easy radiological identification of PPPV.

The first presentation of PPPV was reported in 1972 by Brook et al. [12]. A literature review of relevant articles between 1972 and 2019 was conducted using the MEDLINE (PubMed) database. Only eight cases have been reported. Additionally, relevant articles written in Japanese were searched from Ichushi-web between 1983 and 2019, and only six reported cases were identified. We analyzed 15 cases including ours as shown in Table 1 [7, 9–20]. Eleven patients underwent surgery, including five who underwent PD. Among them, three had carcinoma of the bile duct, and two had ampullary carcinoma. Three patients required PV resection and reconstruction. In one case, pathological invasion to PV was noted. However, in two cases, PV resection was performed despite no evidence of invasion. In contrast, two patients avoided PV resection. In one case, R2 resection was performed to avoid PV resection [9]. In the other case, PV was isolated from the pancreas and resected [17]. However, this report revealed that the shape and thickness of PPPV were almost same as a normal PV. In three PPPV cases, the thinner thickness was confirmed pathologically (approximately 100–200 µm). The fragile thinner wall contributed to the difficulty in isolating the PPPV from the pancreas, duodenum, and bile duct. In two PV resection cases, PPPV isolation was initially attempted. However, the fragile wall of the PPPV caused massive bleeding.

**Table 1:** Fifteen previous cases of PPPV

Refs.	Author	Year	Age	Sex	Diagnosis	Surgical Methods	PPPV Isolation
[12]	Brook W	1972	84	F	Gallstone	Cholecystectomy	–
[9]	Matsumoto Y	1983	64	M	Carcinoma of bile duct	PD	–
[13]	Dumeige F	1989	–	–	Chronic cholecystitis	laparotomy	–
[14]	Matsui N	1995	66	F	Carcinoma of bile duct	PD + PV resection	Fail
[15]	Yasui M	1998	65	M	Cecal cancer	Colon resection	–
[16]	Ozeki Y	1999	62	F	Liver metastasis	Hepatectomy	–
[17]	Tanaka K	2000	61	M	Carcinoma of bile duct	PD	Success
[10]	Inoue M	2003	50	M	Gastric cancer	Gastrectomy	–
[18]	Jung YJ	2005	28	F	cholecystitis	Cholecystectomy	–
[11]	Tomizawa N	2010	74	M	Liver metastasis	None	–
[11]	Tomizawa N	2010	74	F	Breast cancer	None	–
[7]	Jain VK	2013	56	F	Normal	None	–
[19]	Shimizu D	2014	85	F	Ampullary carcinoma	PD + PV resection	Fail
[20]	Goussous N	2017	55	F	Common bile duct stone	ERCP	–
	Our case	2020	73	M	Ampullary carcinoma	PD + PV resection	Fail

Eleven patients underwent surgery, including five who underwent PD. Among them, three had carcinoma of the bile duct, and two had ampullary carcinoma. Three patients required PV resection and reconstruction. In one case, pathological invasion to the PV was noted. However, PV resection was performed despite no evidence of invasion in two cases

For malignant disease such as distal bile duct carcinoma, pancreatic head cancer, and ampullary carcinoma, it is necessary to perform PD (removal of the duodenum, pancreas head, and bile duct). In general, PD with PV resection is related to more surgical complications (Clavien Dindo grade ≥ 3) than PD without PV resection [21]. In fact, PPPV resection can cause postoperative complications such as portal vein thrombosis, hyperammonemia, and intractable ascites. On the other hand, no one has yet to isolate a typical PPPV in the literature. Additionally, aggressive isolation significantly increased the risk of massive bleeding in two cases. Of course, it is certainly better to isolate the PPPV, but it might be safer and more effective to resect and reconstruct a typical PPPV rather than attempt isolation from other organs.

Nakao et al. [22, 23] reported safe PV reconstruction methods using the antithrombogenic Anthron bypass catheter. The catheter is made of a heparinized hydrophilic polymer to allow PV bypass during PV reconstruction. This method could help address some common issues associated with long-term PV obstruction, such as hepatic insufficiency, increased bleeding, and congestion of the small intestine.

For PPPV reconstruction, it might be difficult to perform end-to-end anastomosis given the long distance. Shimizu et al. [19] used the left renal vein as a graft. In our case, both the “normal” PV and PPPV were resected to remove the bile duct and pancreas. As a result, we obtained 3 cm of the “normal” PV without cancer for use as an allograft.

Since PPPV is a rare vascular variant, this study has several limitations. First, owing to the previously reported literature, it was easy to diagnose preoperatively, given the typical L-shaped or inverted L-shaped PV on CT. However, Tanaka et al. [17] reported a normal-shaped PPPV. Second, according to Shimizu, et al. [19] and our case, PV resection rather than isolation represents a safer and more effective alternative if there is no invasion. However, only these two studies reported perioperative events such as operation time, blood loss, and postoperative course in detail. Thus, it was difficult to compare PPPV isolation with PPPV resection. Therefore, we hope that additional studies on this vascular variant will be conducted in the future.

## Data Availability

The datasets used and analyzed during the current study are available from the corresponding author upon reasonable request.

## Abbreviations

HBP: Hepato-biliary-pancreatic
PV: Portal vein
PD: Pancreaticoduodenectomy
PPPV: Prepancreas postduodenal portal vein
AJCC: American Joint Committee on Cancer
SMV: Superior mesenteric vein
SPV: Splenic vein

## References

[1] Atasoy C, Ozyurek E (2006). Prevalence and types of main and right portal vein branching variations on MDCT. AJR Am J Roentgenol.

[2] Ozbayrak M, Tatli S (2016). Cross-sectional imaging of congenital and acquired abnormalities of the portal venous system. Diagn Interv Radiol.

[3] Iqbal S, Iqbal R, Iqbal F (2017). Surgical implications of portal vein variations and liver segmentations: a recent update. J Clin Diagn Res.

[4] Riley DSBM, Kienle GS, Aronson JK, von Schoen AT, Tugwell P, Kiene H, Halfand M, Altman DG, Sox H, Werthmann PG, Moher D, Rison RA, Shamseer L, Koch CA, Sung GH, Hanaway P, Sudak NL, Kaszkin-Bettag M, Carpenter JE, Gagnier JJ (2017). CARE guidelines for case reports: explanation and elaboration document. J Clin Epidemiol.

[5] Amin MBES, Greene F, Byrd DR, Brookland RK, Washington MK, Gershenwald JE, Compton CC, Hess KR, Sullivan DC, Jessup JM, Brierley JD, Gaspar LE, Schilsky RL, Balch CM, Winchester DP, Asare EA, Madera M, Gress DM, Meyer LR (2017). AJCC Cancer Staging Manual.

[6] Zhao ZL, Wei Y, Wang TL, Peng LL, Li Y, Yu MA (2020). Imaging and pathological features of idiopathic portal hypertension and differential diagnosis from liver cirrhosis. Sci Rep.

[7] Jain VK, Rajesh S, Bhatnagar S, Dev A, Mukund A, Arora A (2013). Prepancreatic postduodenal portal vein: a rare vascular variant detected on imaging. Surg Radiol Anat.

[8] Marks C (1969). Developmental basis of the portal venous system. Am J Surg.

[9] Matsumoto Y, Sugahara K, Ida T, Mashimo R, Wen H, Fujii H (1983). Anomalies of the portal venous system: pathogenesis and its surgical implications. Jpn J Gastroenterol Surg.

[10] Inoue M, Taenaka N, Nishimura S, Kawamura T, Aki T, Yamaki K (2003). Prepancreatic postduodenal portal vein: report of a case. Surg Today.

[11] Tomizawa N, Akai H, Akahane M, Ino K, Kiryu S, Ohtomo K (2010). Prepancreatic postduodenal portal vein: a new hypothesis for the development of the portal venous system. Jpn J Radiol.

[12] Brook W, Gardner M (1972). Anteroposition of the portal vein and spontaneous passage of gall-stones. Case report and embryological hypothesis. Br J Surg.

[13] Dumeige F, Hermieu JF, Farret O (1989). A pre-wirsungal portal vein. Apropos of a case. Chirurgie.

[14] Matsui N, Morita T, Harada M, Morikage N, Kanazawa M, Nakamura T (1995). A case of carcinoma of the bile duct with anomaly of the portal venous system- parepancreatic postduodenal portal vein. Jpn J Gastroenterol Surg.

[15] Yasui M, Tsunoo H, Nakahara H, Asano M, Fujita H (1998). Portal veinpositioned anterior to the pancreas and posterior to the duodenum-report of a case. J Jpn Surg Assoc.

[16] Ozeki Y, Tateyama K, Sumi Y, Yamada T, Yamauchi K, Bandoh M (1999). Major hepatectomy for liver tumor with anomalous portal branching. Jpn J Gastroenterol Surg.

[17] Tanaka K, Sano K, Yano F, Ohhira Y, Takahashi T, Suda K (2000). A case of carcinoma of the inferior bile duct with anomaly of the portal venous system-prepancreatic postduodenal portal vein. Operation.

[18] Jung YJLS, Yang SB, Park WK, Chang JC, Kim JW (2005). Prepancreatic postduodenal portal vein: a case report. J Korean Radiol Soc.

[19] Shimizu D, Fujii T, Suenaga M, Niwa Y, Okumura N, Kanda M (2014). A case of the ampulla of vater with anomaly of the portal venous system: prepancreatic postduodenal portal vein. Jpn J Gastroenterol Surg.

[20] Goussous N, Cunningham SC (2017). Prepancreatic postduodenal portal vein: a case report and review of the literature. J Med Case Rep.

[21] Klein FBF, Felsenstein M, Malinka T, Pelzer U, Denecke T, Pratschke J, Bahra M (2018). Routine portal vein resection for pancreatic adenocarcinoma shows no benefit in overall survival. Eur J Surg Oncol.

[22] Nakao A, Horisawa M, Suenaga M (1982). Temporal portsystemic bypass with the use of the heparinized hydrophilic catheter. Jpn J Artif Organs.

[23] Nakao A, Nonami T, Harada A, Kasuga T, Takagi H (1990). Portal vein resection with a new antithrombogenic catheter. Surgery.

